# HOW CAN WE PROTECT PEAK BONE MASS AND FUTURE BONE HEALTH FOR ADOLESCENT WOMEN? - BY SUPPORTING OVULATION AND AVOIDING COMBINED HORMONAL CONTRACEPTION USE

**DOI:** 10.1590/1984-0462/;2017;35;2;00019

**Published:** 2017

**Authors:** Jerilynn Celia Prior

**Affiliations:** a Centre for Menstrual Cycle and Ovulation Research, Endocrinology and Metabolism [Centro para Pesquisa sobre Ciclo Menstrual e Ovulação, Endocrinologia e Metabolismo], University of British Columbia, Vancouver, Canadá.

The importance of achieving optimal peak bone mass (PBM) is well accepted, given each young person’s heredity, intra-uterine supportive system, social, nutritional, and physical environments.^1^ The belief is that “an increase of PBM by one standard deviation would reduce the fracture risk by 50%,”^1^ although this has yet to be proven prospectively. We also understand the importance of a normal age at menarche for PBM of women, and to decrease later life hip^2^ and spine fracture risks. Population-based prospective data show that PBM at the femoral neck and total hip, by a real bone mineral density (BMD), are achieved between ages 16 and 19 years, with spinal PBM occurring somewhat later.^3^

New information indicates that achieving an ovulatory cycle is important for the PBM of the whole body,^4^ and for maintaining spinal PBM through the menstruating years.^5^ Thus it is important to assess adolescent menstrual cycles for ovulatory status and to physiologically treat (with cyclic progesterone) menstrual cycle-related disturbances (oligo-/amenorrhea, cramps, heavy flow, and acne). Recent evidence shows that, at least in North America, menstrual cycle-related disturbances are often “treated” with combined hormonal contraception (CHC)^6^ ^,^ ^7^ (which could include oral, transdermal, or vaginal agents), despite the fact that CHC use is increasingly associated with PBM interference.^8^ ^,^ ^9^

## NORMAL CYCLE AND OVULATION DEVELOPMENT

During the first year following menarche, menstrual cycles are normally irregular and often far apart.^10^ Most of us assume that, by the time adolescent cycles become regular (although they may be as long as 41 days apart), they are also normally ovulatory. Maturation to predictably ovulatory cycles with a normal luteal phase length, however, takes about 12 years after menarche.^10^ Many things that are common for adolescent women today may interfere with the development of normally ovulatory cycles. These include worry about weight, being bullied, experiencing stigma, or any nutritional or psychosocial stressors. Ensuring that adolescents experience a sense of achievement (can be scholastic, in sports, in hobbies, or almost anything), are accepted by at least a few of their peers, and feel the affection of some close family or others, is necessary for normal reproductive as well as for emotional (and likely bone) health.

## OVULATORY CYCLES, PEAK BONE MASS, AND BONE HEALTH

**Figure 1:**
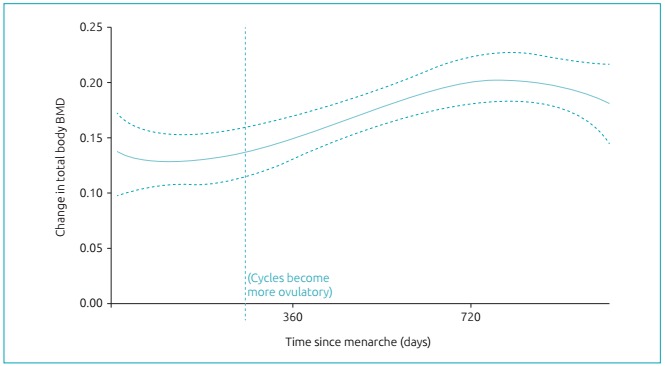
Regression analysis of change in whole body bone mineral density (BMD) over three years in 13 adolescent women who collected salivary progesterone levels from menarche onwards at three weeks after flow and weekly thereafter until menstrual flow begin.

Does bone change relate to maturation in ovulation development as well as to regular cycles? This has not been well investigated, but one small prospective study showed ovulation took at least 10 months post-menarche to first begin^4^ ^,^ and even more than menarche, was temporally associated with whole body BMD gain (Figure 1).^4^ Furthermore, a meta-analysis of women from their teens through their 30s, showed that those with more versus fewer ovulation-disturbed cycles had almost one percent per year more negative spinal BMD changes (-0.86%/y [95%CI -1.68--0.04] *p*=0.04).^5^ Why? Because the normal increase and decrease of estradiol levels within each menstrual cycle causes some bone resorption that progesterone can counterbalance by stimulating osteoblastic bone formation.^11^

## TREATMENT OF TEEN MENSTRUAL CYCLE-RELATED DISTURBANCES

Menstrual cramps (dysmenorrhea) are common in adolescent women and effectively treated by intense anti-prostaglandin therapy staying ahead of the pain^12^ ^.^ Acne is also prevalent in young women around menarche, gets better with time, and can usually be controlled by avoiding facial oil-based exposures, eating a healthy diet, and use of over-the-counter topical drying agents. A few women will get very heavy flow related to estrogen excess and an ovulation. As mentioned, irregular cycles are the norm for at least the first post-menarche year, and a few normal young women will skip cycles for months at a time for several more years. But these normal maturational issues are often inappropriately “treated” with combined hormonal contraception^13^. As pharmacological doses of synthetic estrogen and progestin, CHC causes regular withdrawal flow but actually “covers up” rather than ­facilitating reproductive maturation or resolving the underlying issue.

Most of the cycle, flow, cramps, and skin-related problems of adolescents are related to an imbalance: too much estrogen and too little progesterone. Therefore, cyclic progesterone therapy (oral micronized progesterone, 300 mg at bedtime for 14 days/cycle) is an ideal initial or transitional treatment. However, this notion has only been scientifically tested as cyclic medroxyprogesterone (10 mg for 10 days/month) for hypothalamic amenorrhea, oligomenorrhea, regular cycles with anovulation or short luteal phases in normal-weight women ages 20-35 years,^14^ in whom it caused a significant increase in spinal BMD (+2.0%/y *versus* -2.0%/y in placebo). My clinical experience is that cyclic progesterone plus social, emotional, and nutritional support is highly effective for maturation of both ovulation and bone.

## COMBINED HORMONAL CONTRACEPTION USE AND ACHIEVING AND MAINTAINING PEAK BONE MASS

Increasing evidence shows that use of CHC during adolescence may be related to less positive gain to PBM^13^. This may occur because the supra-physiological dose of ethinyl estradiol (needed to prevent pregnancy), suppresses bone modeling that is necessary to achieve PBM. Furthermore, a recent random effects meta-analysis showed that more negative rates of two-year spinal BMD change (-0.02 [95%CI -0.03--0.01] g/cm^2^; *p*=0.0007) occurred for ~900 women ages 12-19 years, using CHC versus non-using controls (Goshtasebi, 2017, submitted). These are yet a further reason to use cyclic progesterone therapy^15^ rather than CHC for symptomatic adolescents with “funny cycles,” cramps, acne, or heavy flow.

## CONCLUSION

Adolescent maturation requires increased attention, although we are all aware that adolescence is a time of growth and maturation. Almost all cycle-related problems in adolescents seem to be reflexly treated with CHC, meaning with high-dose, suppressive, exogenous hormones. In particular, we need to carefully examine adolescent maturation related to the reproductive and musculoskeletal systems. With the perspective that there is a unique, once-in-a-lifetime window of opportunity to develop normally ovulatory cycles and optimal PBM, disturbances of these need to first be detected, and then treated physiologically. Cyclic progesterone treatment versus CHC, however, still requires randomized, controlled, trial examination for its effects on adolescent reproductive problems and bone change. We must exercise caution before prescribing CHC for adolescent problems, given that other treatments are effective, and for birth control, given that other options for heterosexually active teens at risk of pregnancy are also available.^16^

## References

[1] Bonjour JP, Chevalley T, Ferrari S, Rizzoli R (2009). The importance and relevance of peak bone mass in the prevalence of osteoporosis. Salud Publica Mex.

[2] Johnell O, Gullberg B, Kanis JA, Allander E, Elffors L, Dequeker J (1995). Risk factors for hip fracture in European women: the MEDOS Study. Mediterranean Osteoporosis Study. J Bone Miner Res.

[3] Berger C, Goltzman D, Langsetmo L, Joseph L, Jackson S, Kreiger N (2010). Peak bone mass from longitudinal data: implications for the prevalence, pathophysiology, and diagnosis of osteoporosis. J Bone Miner Res.

[4] Kalyan S, Barr SI, Alamoudi R, Prior JC (2007). Is Development of Ovulatory Cycles in Adolescence Important for Peak Bone Mass?. J Bone Miner Res.

[5] Li D, Hitchcock CL, Barr SI, Yu T, Prior JC (2014). Negative Spinal Bone Mineral Density Changes and Subclinical Ovulatory Disturbances--Prospective Data in Healthy Premenopausal Women With Regular Menstrual Cycles. Epidemiol Rev.

[6] Jones RK (2011). Beyond birth control: the overlooked benefits of oral contraceptive pills.

[7] Chen R, Bejaei F, Shan Y, Vali T, Prior JC (2016). Health care provider hormonal recommendations for treatment of menstrual-cycle related problems - a vignette-based study. Br J Pharmecutical Res.

[8] Polatti F, Perotti F, Filippa N, Gallina D, Nappi RE (1995). Bone mass and long-term monophasic oral contraceptive treatment in young women. Contraception.

[9] Berenson AB, Rahman M, Breitkopf CR, Bi LX (2008). Effects of depot medroxyprogesterone acetate and 20-microgram oral contraceptives on bone mineral density. Obstet Gynecol.

[10] Vollman RF, Friedman EA (1977). The menstrual cycle. Major Problems in Obstetrics and Gynecology.

[11] Seifert-Klauss V, Prior JC (2010). Progesterone and bone: Actions promoting bone health in women. J Osteop.

[12] CeMCOR - The Centre for Menstrual Cycle and Ovulation Research Painful Periods.

[13] Prior JC (2016). Adolescents’ use of combined hormonal contraceptives for menstrual cycle-related problem treatment and contraception: evidence of potential lifelong negative reproductive and bone effects. Womens’s Reproduct. Health.

[14] Prior JC, Vigna YM, Barr SI, Rexworthy C, Lentle BC (1994). Cyclic medroxyprogesterone treatment increases bone density: a controlled trial in active women with menstrual cycle disturbances. Am J Med.

[15] CeMCOR - The Centre for Menstrual Cycle and Ovulation Research Cyclic Progesterone Therapy.

[16] CeMCOR - The Centre for Menstrual Cycle and Ovulation Research Contraception.

